# Activators of alpha synuclein expression identified by reporter cell line-based high throughput drug screen

**DOI:** 10.1038/s41598-021-98841-9

**Published:** 2021-10-06

**Authors:** Fabian Stahl, Philip Denner, Dominik Piston, Bernd O. Evert, Laura de Boni, Ina Schmitt, Peter Breuer, Ullrich Wüllner

**Affiliations:** 1 DZNE, German Center for Neurodegenerative Diseases, Venusberg-Campus 1/99, 53127 Bonn, Germany; 2 Department of Neurology, University Hospital Bonn, 53127 Bonn, Germany

**Keywords:** Drug discovery, Diseases

## Abstract

Multiplications, mutations and dysregulation of the alpha synuclein gene (*SNCA*) are associated with the demise of dopaminergic neurons and are considered to play important roles in the pathogenesis of familial and sporadic forms of Parkinson’s disease. Regulation of *SNCA* expression might thus be an appropriate target for treatment. We aimed to identify specific modulators of *SNCA* transcription, generated CRISPR/Cas9 modified *SNCA*-*GFP*-luciferase (*LUC*) genomic fusion- and control cell lines and screened a library of 1649 bioactive compounds, including the FDA approved drugs. We found no inhibitors but three selective activators which increased *SNCA* mRNA and protein levels.

## Introduction

α-Synuclein (α-syn) is a key component in familiar and sporadic Parkinson’s disease (PD) pathophysiology. Point mutations in the *SNCA* gene and multiplication of wildtype *SNCA* cause familiar parkinsonian syndromes. Increased α-syn protein levels correlate with the severity of symptoms^1^. These gene-dosage effects suggest that *SNCA* mRNA levels are a relevant target to be addressed. Several modifier screens (genetically or compound modifiers) for α-syn induced toxicity based on α-syn overexpression-models have been performed in different organisms like yeast, *E. coli*, *C. elegans,* in rodent- and human cell lines. In these approaches, measuring cell growth and/or cell viability served as readout for presumed α-syn protein toxicity. Despite the large number of studies, unbiased screens in human derived cell lines had been scarce^2^. α-Syn overexpression screens do not account for a regulation of endogenous *SNCA*. Thus, genes or compounds which modulate the epigenetic and transcriptional landscape might have been missed. Mittal and colleagues^3^ performed the first study addressing endogenous *SNCA* mRNA expression by screening a library of FDA approved compounds and found that β2 adrenoreceptor (β2AR) agonists reduced *SNCA* mRNA and α-syn protein levels. We had chosen an alternative approach to identify modifiers of *SNCA* expression and designed a luciferase (*LUC*) reporter-based high throughput screening of 1649 bioactive drugs including 845 FDA approved compounds in CRISPR/Cas9 modified human SH-SY5Y neuroblastoma cell lines. We identified three selective activators of *SNCA* mRNA and α-syn protein levels.

## Results

### Reporter cell line-based screening of 1649 bioactive compounds

**Figure 1:**
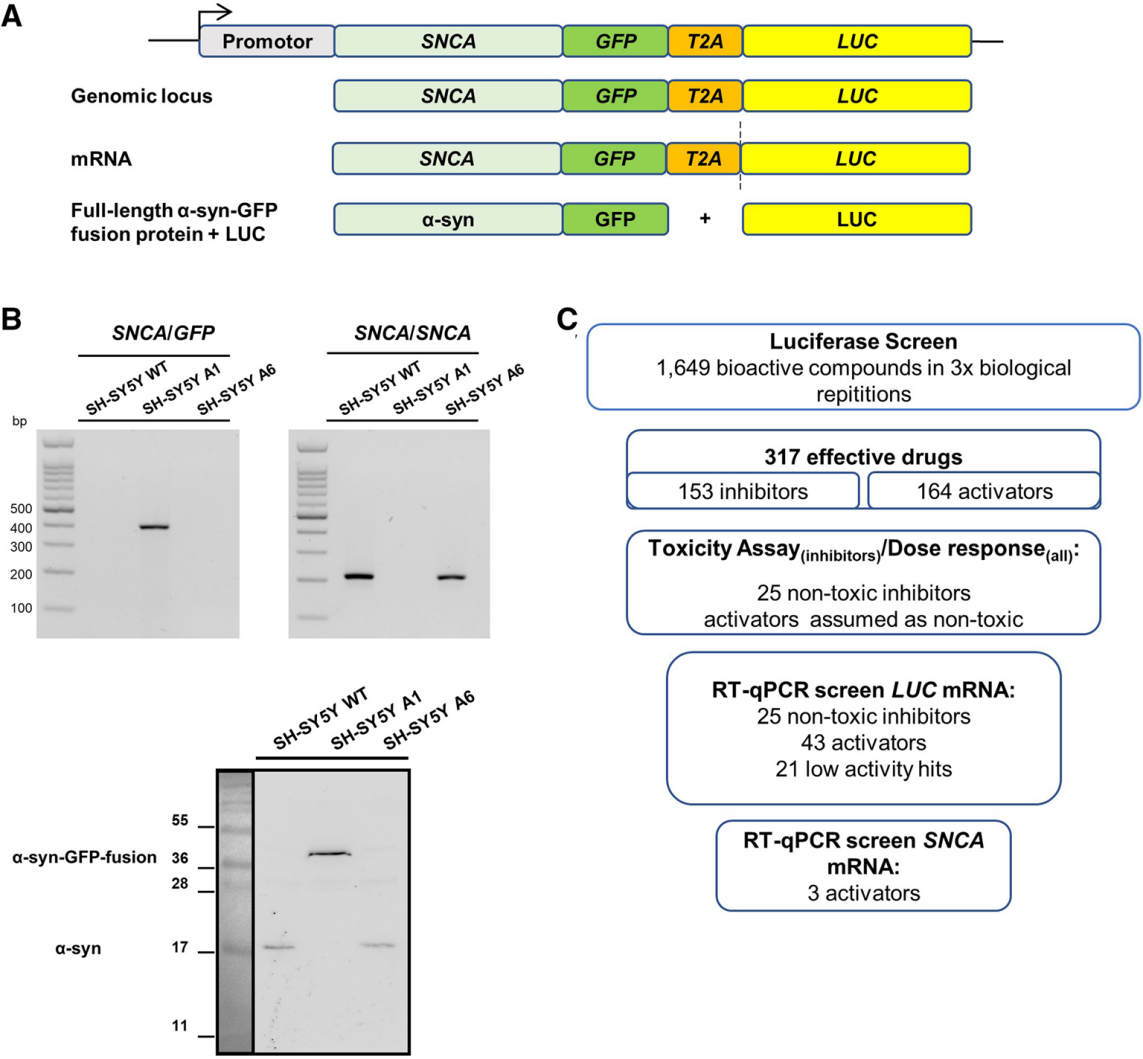
*SNCA*-*GFP*-*LUC* reporter cell line-based high throughput screening assay of 1649 FDA approved drugs identifies modulators of α-syn mRNA and protein levels. (**A**) Schematic overview of CRISPR/Cas9 generated, genomic *SNCA-GFP-LUC* fusion under control of the endogenous *SNCA* promotor in the SH-SY5Y cell line. Translation of its mRNA results in both, α-syn-GFP fusion protein and a functional LUC protein, separated by a ribosome skipping event due to the *T2A* sequence. (**B**) Agarose gel-electrophoresis of PCR from cDNA (top) and Western blot (bottom) of the reporter cell line confirming in-frame *SNCA*-*GFP*-*LUC* fusion at mRNA- and α-syn-GFP at protein level, respectively. (**C**) Workflow of the screening and validation procedure of α-syn modulating drugs. Several exposure images of full-size WB in (**B**) (lower panel) see Supplementary Fig. S2.

A CRISPR/Cas9 *SNCA*-*GFP*-*T2A*-*LUC* fusion cell line (A1) expressing α-syn-GFP and LUC under the control of the endogenous human *SNCA* promoter was generated to identify modulators of *SNCA* expression (Fig. 1A). Cell lines with random integration of the reporter construct (A6) were selected as control for unspecific modulators of gene expression (example of unspecific modulators see Supplementary Fig. S1). Proper integration of the constructs was analyzed by PCR (Fig. 1B, upper panel) and Western blot (Fig. 1B, lower panel).

**Figure 2:**
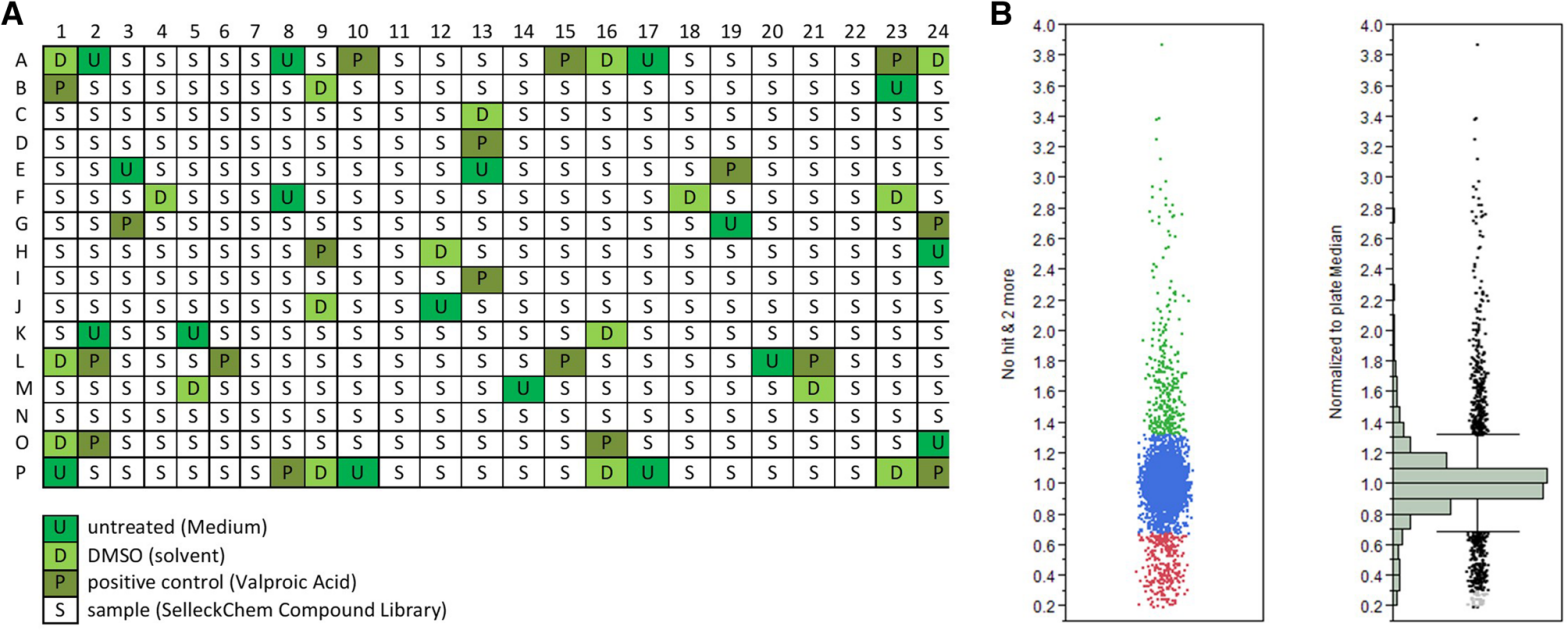
Plate layout and quality control of the luciferase reporter drug screening assay. (**A**) Plate layout of randomly distributed drugs and controls. (**B**) Threshold for hit definition: compounds were considered as effective when showing a difference beyond the four-fold standard deviation (4 × SD = 0.33) compared to untreated control.

Three independent experimental repetitions revealed 153 potentially inhibiting and 164 activating compounds; 1322 compounds were within the four-fold SD cut off or without any effect (Figs. 1C and 2B).

To exclude cytotoxic effects of potential inhibitors, a cell viability test comprising a homogenous resazurin- and an image based high content screen (HCS) on single cell level were performed in a dose range from 250 nM to 40 µM. We tested the 94 most potent inhibitors while all activators were considered as non-toxic. Sixty-nine potential inhibitors were identified as toxic for our cells and were omitted from subsequent experiments (workflow see Fig. 1C).

### Compounds modulating *LUC* and *SNCA* mRNA and α-syn protein expression levels

**Figure 3:**
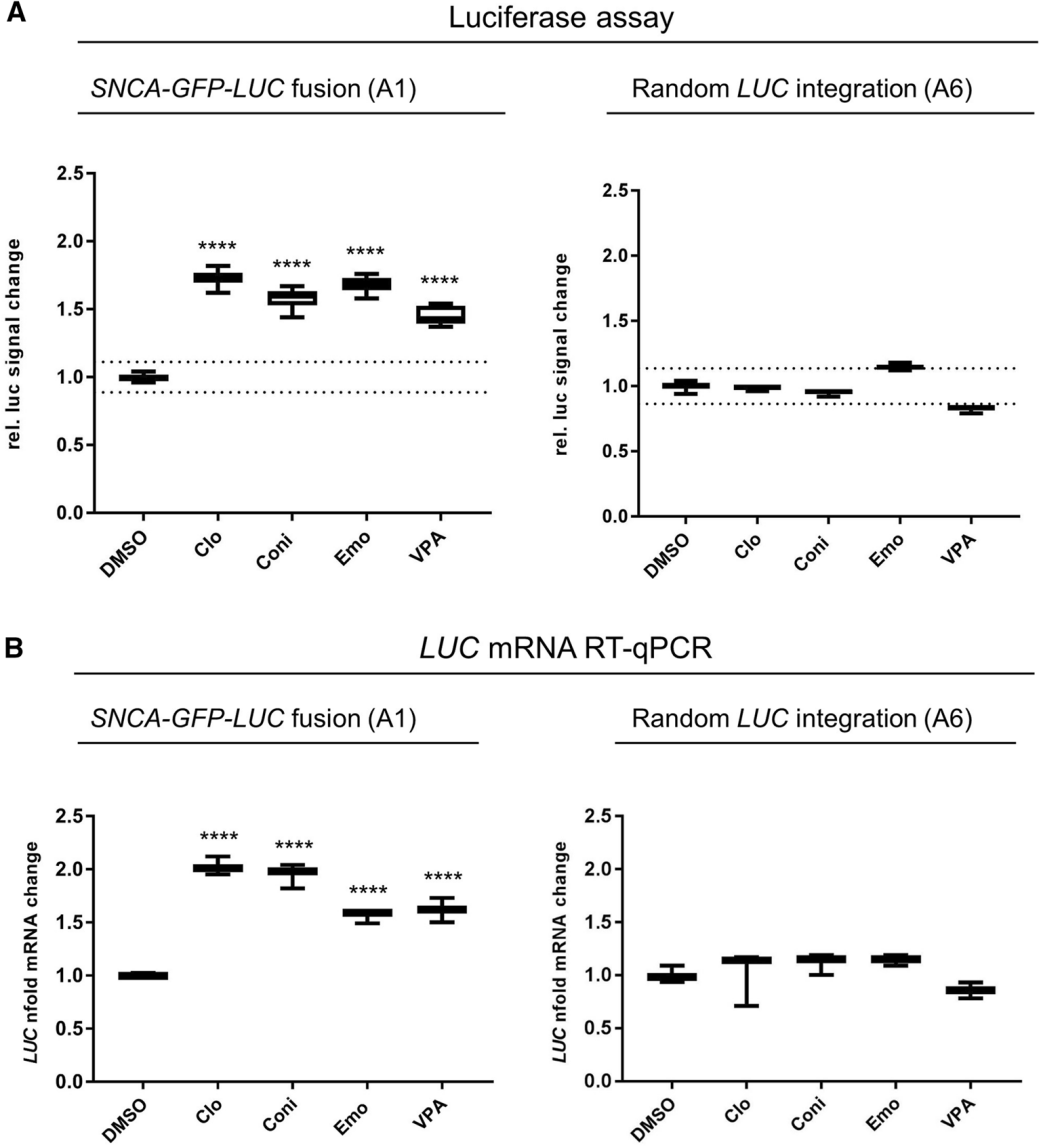
LUC assay and *LUC* RT-qPCR in the screening (A1) and control cell line (A6) for activators of α-syn. (**A**) Signal fold-change in the LUC assay of A1 and A6 cell lines and (**B**) expression fold changes of *LUC* mRNA measured with RT-qPCR. LUC signal change was determined by normalizing six replicates of treated cells to DMSO. RT-qPCR data were normalized to three housekeeping genes and DMSO control, in triplicates, respectively. Boxplot diagrams represent 5–95 percentile. Dotted lines in (**A**) depicts threshold of four-fold standard deviation of DMSO control. Compounds were applied at a final concentration of 25 µM (Clo 12.5 µM) for 24 h. VPA was used as a positive control at a concentration of 600 µM. ****p = 0.0001.

To corroborate that the observed changes in LUC derived chemiluminescence were indeed due to altered *LUC* gene expression, we performed additional RT-qPCR analysis. Quantifying the *LUC* mRNA in an intermediate step allowed the direct comparison between the A1 screening- and A6 counter-screening cell line to exclude compounds inducing rather unspecific gene expression changes (Fig. 3).

Thus, the 25 non-toxic inhibitors, 43 activators and 21 low activity hits were investigated by RT-qPCR. None of the putative non-toxic inhibitors reduced *LUC* mRNA levels at the *SNCA* locus, whereas three compounds increased *LUC* mRNA specifically in the A1 cell line. These compounds were also tested for pleiotropic modulation of transcription in the A6 control cell line and found to be sufficiently specific for *SNCA* (Fig. 3).

**Figure 4:**
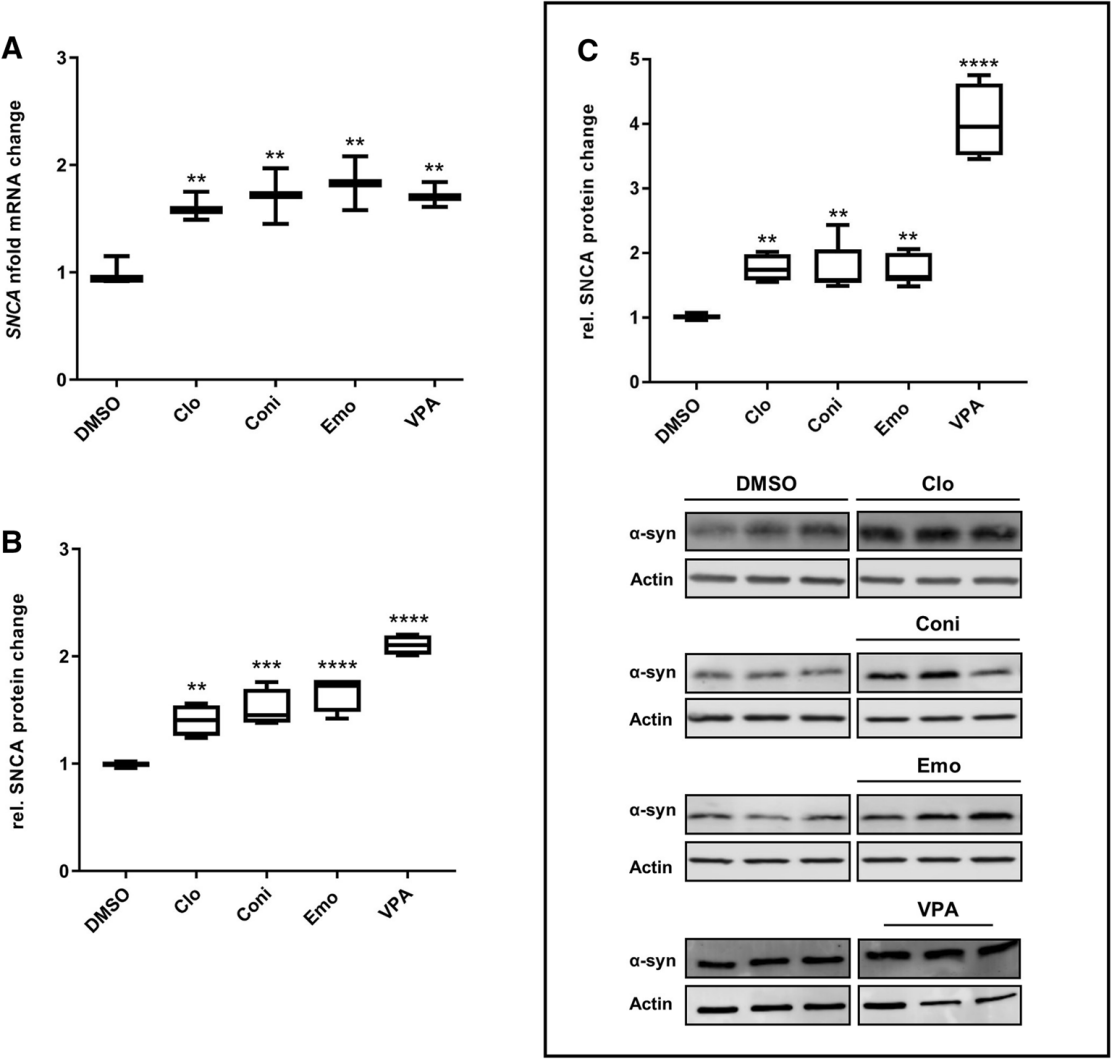
Compound treatment increased α-syn mRNA and protein levels in SH-SY5Y wildtype cells. (**A**) Expression fold change of *SNCA* mRNA measured with RT-qPCR. (**B**) Protein levels were determined by ICW and (**C**) WB in SH-SY5Y wild type cells. RT-qPCR data were normalized to three housekeeping genes and DMSO control, in triplicates, respectively. The Boxplot diagrams for (**B**) ICW represent the normalized mean of four 96-well plates with six to seven repetitions per compound, respectively. (**C**) For WBs six (VPA n = four) repetitions were conducted. Boxplot diagrams represent 5–95 percentile. (**C**, lower) Representative WB. Compounds were applied at a final concentration of 25 µM (Clo 12.5 µM) for 24 h. VPA was used as a positive control at a concentration of 600 µM. *p < 0.1 **p < 0.01, ***p < 0.001, ****p = 0.0001. Full-size WB of DMSO and Clo see Supplementary Fig. S4A, DMSO and Coni see Supplementary Fig. S4B, DMSO and Emo see Supplementary Fig. S4C and DMSO and VPA see Supplementary Fig. S4D. ICWs (**B**) and WB (**C**) membranes were imaged with the LI-COR Odysseys Clx (Model 9140; S/N CLX-0554) and signals were quantified using the Image Studio software 4.0.21.

We finally determined *SNCA* mRNA expression levels in native non-modified SH-SY5Y wildtype cells and verified three compounds increasing *SNCA* mRNA levels 1.4 to twofold compared to DMSO control (Fig. 4A): clomiphene-citrate (Clo), a selective estrogen receptor modulator (SERM), conivaptan-HCL (Coni) a vasopressin receptor antagonist and the anthraquinone emodin (Emo).

To assess whether increased *SNCA* mRNA transcripts result in potentially relevant increases in α-syn protein amount, we performed two independent protein assays: in-cell Western (ICW) (example ICW see Supplementary Fig. S3) and conventional Western blot (WB). Both approaches revealed that treatment with the *SNCA* activators resulted in a 1.3 to 2-fold increase of α-syn protein levels compared to DMSO control samples (Fig. 4B,C). The relative changes in protein level of ICW and WB, corresponded to the observed *SNCA* mRNA changes in the previous RT-qPCR assay.

### Effects on histone modification and DNA methylation in SH-SY5Y wild type cells

**Figure 5:**
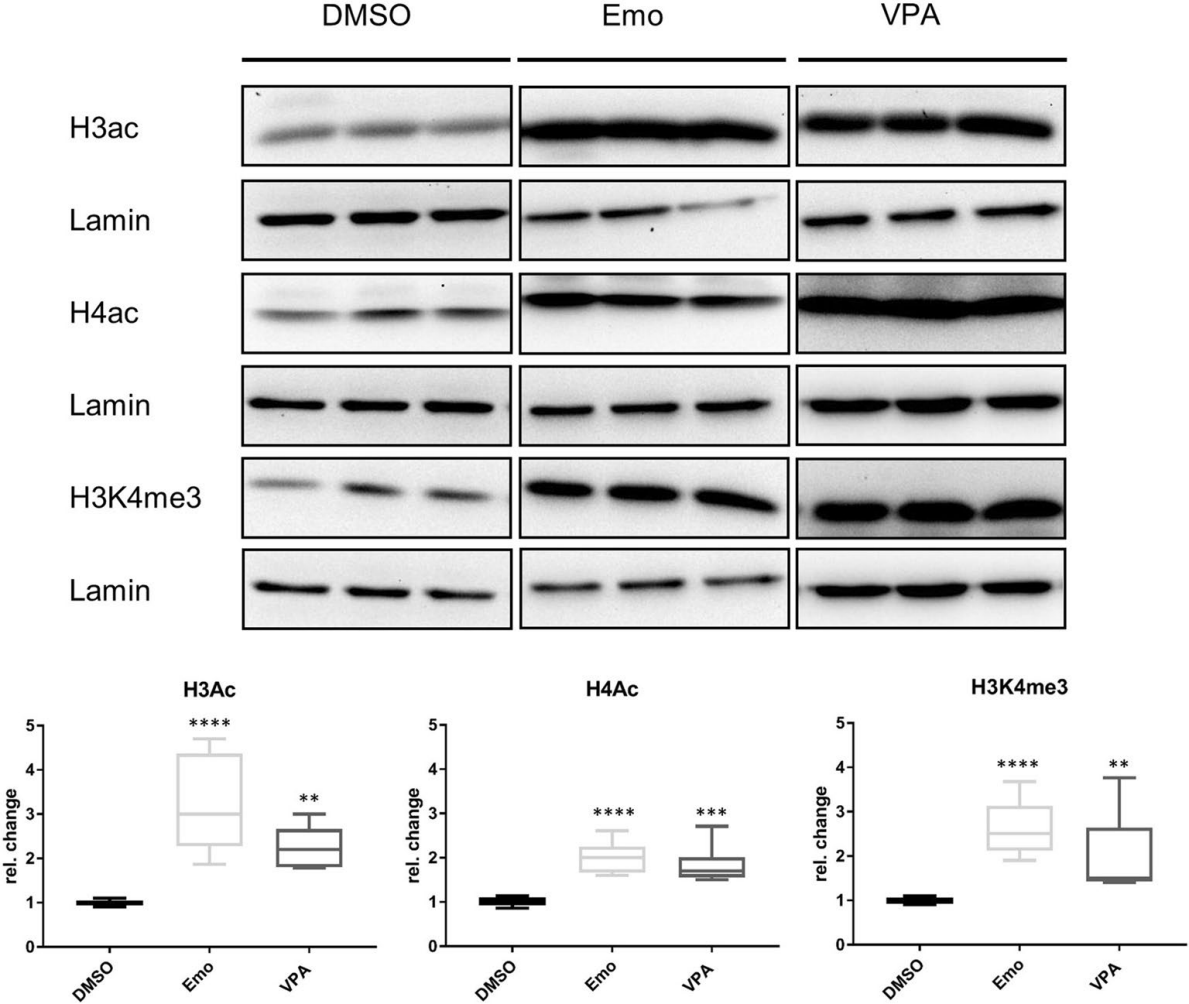
Emo increased histone H3/H4ac and H3K4me3 levels. Representative WB (upper) and boxplot diagrams (lower) represent 5–95 percentile. Three independent experiments with three repetitions were conducted. Protein levels were determined by Western blot and normalized to Lamin B1 and DMSO, respectively. Compounds were applied at a final concentration of 25 µM for 24 h. VPA was used as a positive control at a concentration of 600 µM. **p < 0.01, ***p < 0.001 ****p = 0.0001. Full-size WBs of H3ac for DMSO, Emo and VPA see Supplementary Fig. S5A,B. Full size WBs of H4ac for DMSO, Emo and VPA see Supplementary Fig. S5C,D, and WBs of H3K4me3 see Supplementary Fig. S5D (VPA) and E (DMSO, Emo).

To investigate, whether chromatin alterations were involved in the observed *SNCA* mRNA increases, we analyzed global acetylation at histone H3 and H4 (H3/H4ac) and H3K4 tri-methylation (H3K4me3). We found that Emo consistently increased H3/H4ac and H3K4me3 marks (Fig. 5, lower). Clo and Coni showed no significant association with histone methylation or acetylation levels (see Supplementary Fig. S5F). Also, no differences in *SNCA* intron 1 DNA methylation^4^ were observed after treatment with either compound (see Supplementary Fig. S6).

## Discussion

We performed a *LUC* reporter-based high throughput screen (HTS) and subsequent RT-qPCR assays to screen a library of 1649 bioactive compounds for transcriptional modifiers of *SNCA* expression. Initially, 163 potential activators and 153 potential inhibitors were identified. After cell viability assessment, we selected 25 inhibitors, 43 activators and 21 low activity hits for further characterization (Fig. 1C). To exclude non-transcriptional modulators and compounds confounding the *LUC* readout, RT-qPCR assays were performed^5,6^. Indeed, among the 25 potential inhibitors we found none which reduced *SNCA* mRNA levels, but identified three compounds which specifically increased *SNCA* mRNA and α-syn protein levels (Figs. 3, 4).

The absence of a clear-cut inhibitor is in contrast to the work of Mittal and colleagues, who quantified mRNA levels of endogenous *SNCA* in SK-N-MC neuroblastoma cells. They discovered the selective β2 adrenoreceptor (β2AR) agonist metaproterenol to reduce *SNCA* mRNA and α-syn protein levels^3,6^. Our screening approach included 47 adrenergic receptor modulators but none of the tested modulators (agonists and antagonists, including metaproterenol) were active in the primary *LUC* assay. A literature search and the online database “Human Protein Atlas” (data available from: https://www.proteinatlas.org/ENSG00000169252-ADRB2/cell)^7^ revealed that β2AR are not expressed in SH-SY5Y cells. Indeed, WB did not detect β2AR expression in our SH-SY5Y cells (see Supplementary Figs. S7, S8). We had selected the SH-SY5Y neuroblastoma lineage, which is frequently chosen to model PD, because of human origin, catecholaminergic neuronal properties and the ease of genetic engineering—very similar to SK-N-MC^8^. Clearly, the diverging results call for an even more conscientious selection of the screening cells. Induced human pluripotent stem cells (iPSC) or thereof derived cells are closer to the actual neurons in human brain and may offer an alternative, although these cells are difficult to standardize on the other hand.

The three selective activators comprised hormone receptor interacting drugs, i.e. the selective estrogen receptor modulator (SERM) clomiphene-citrate (Clo) and the vasopressin receptor antagonist conivaptan-HCL on the one hand and the plant anthraquinone emodin (Emo) on the other.

Clo was the only SERM in the library which activated *SNCA* mRNA expression. Depending on the target tissue, Clo acts as an estrogenic agonist or antagonist but its precise mechanism is still unknown (https://go.drugbank.com/drugs/DB00882)^9^. Clo is a mixture of the two isomers zuclomiphene (cis-) and enclomiphene (trans-isomer) which show estrogen agonistic and antagonistic effects, respectively (https://drugs.ncats.io/drug/1HRS458QU2). We therefore investigated the effect of zuclomiphene and enclomiphene in our cell lines. Both isomers lead to similar increases of *SNCA* mRNA and no stereo-selectivity was identified (see Supplementary Fig. S9).

Interestingly, estrogenic effects have been reported for the plant derived compound Emo^10^.

The screening library contains a wide spectrum of other SERMS (like bazedoxifene-HCl and toremifene-citrate), ER antagonists (like fulvestrant and raloxifene) and several drugs affecting the estrogen/progesterone receptor pathway (among others aromatase inhibitors, progesterones, progestins, estradiol and its derivates). Raloxifene was among the inhibitors identified in the *LUC* reporter assay but showed no consistent effect in the RT-qPCR assay. Thus, canonical estrogenic effects were considered unlikely for the observed transcriptional modulation of *SNCA*.

Similarly, three vaptanes were tested in the HTS but only Coni the vasopressin V_1A_ and V_2_ receptor antagonist (https://go.drugbank.com/drugs/DB00872)^9^ modulated *SNCA* mRNA and α-syn protein levels. Likely, the observed increase was not related to a vasopressin receptor (V_1A_, V_2_) mediated effect, as vasopressin (10 µM) alone did not show any effect to *SNCA* mRNA levels (see Supplementary Fig. S10).

Epigenetic modifications, i.e. DNA methylation and histone modifications have emerged as important regulators of (*SNCA*) gene expression in PD^11,12^. To date, several findings revealed that posttranslational histone modifications can lead to altered expression levels of *SNCA*. Treatment with valproic acid (VPA), a known histone deacetylase inhibitor (HDAC), induces hyperacetylation of global histone H3 at the *SNCA* promotor and leads to an increase of Snca in rat cerebral granule cells, cortex and cerebellum^13^. Additionally, VPA was shown to increase *SNCA* mRNA and α-syn protein levels in SH-SY5Y cells^14^. Vice versa, reduced H3K27 acetylation marks across the *SNCA* promotor resulted in decreased *SNCA* mRNA levels^3^.

Guhathakurta and colleagues found the transcription promoting mark H3K4me3 significantly enriched at the *SNCA* promotor and intron1 region of substantia nigra in post-mortem PD brain samples. Furthermore, directed de-methylation of H3K4me3 at the *SNCA* promotor decreased *SNCA* mRNA and protein levels in SH-SY5Y cells and idiopathic PD-iPSC^15^.

A recent genome-wide study compared the overall histone acetylation levels in the PD brain and controls. The findings implicated that hyperacetylation of H3K27 is a general phenomenon within PD brains and 24 of the 83 genes bearing hyperacetylated regions of H3K27—including *SNCA*—were marked as risk genes for PD^16^.

Among the screened activators of *SNCA* expression and protein level, Emo led to a significant increase of histone marks for open chromatin, i.e. H3K4me3 and global H3/H4ac levels, similar to VPA (Fig. 5). Our findings are in accordance to earlier studies which found Emo to exhibit HDAC inhibitory function in recombinant HDAC activity assay performed in bovine cardiac tissue^17^. Interestingly, no increased *LUC* signals or mRNA levels were observed for Emo and VPA in the control cell line (A6), which was to be expected if global histone modifications were effective. Emo and emodin-rich rhubarb, however, have been shown to exert gene expression changes similar to the well-established pan-HDAC inhibitor trichostatin A (also part of the screening library)^18^.

Among the 23 known “canonical” HDAC inhibitors in the screening library only Rocilinostat (ACY-1215) was found to increase LUC signal but was excluded from further analysis because it did not meet the quality control criteria. Thus, it seems rather unlikely that increased global histone acetylation alone lead to elevated α-syn levels. It remains to be determined whether Emo and VPA induced histone modifications might be prerequisites for more specific downstream regulations at the *SNCA* promotor.

Compared to the observed mutations^19,20^ and multiplications^1,21^ of the *SNCA* gene in familial PD, the role of *SNCA* in sporadic PD seems to be more elusive. Previous studies have shown conflicting data regarding the expression levels of *SNCA* mRNA in idiopathic PD (iPD). While studies found no alterations of *SNCA* mRNA expression in laser captured dopaminergic neurons from *postmortem* substantia nigra and blood samples from iPD patients and healthy controls^22,23^ another study reported decreased *SNCA* mRNA levels in the substantia nigra-, frontal- and temporal cortex neurons^24^. Increased levels of *SNCA* mRNA were observed in UV-laser micro dissected human *postmortem* substantia nigra neurons and mid-brain tissue including the substantia nigra from iPD patients compared to controls^25,26^.

Our unbiased reporter cell line-based screening of 1649 bioactive and FDA approved compounds did not reveal a substance with an immediate translational value for the modulation of *SNCA* expression. Since we found no specific mode of action for the identified activators (except global histone modifications for Emo) future studies will be needed to uncover potential mechanisms and to evaluate the utility for translational application of these compounds.

Intriguingly, Emo and other anthraquinone-type analogs, like aloe-emodin and emodic acid, have been associated with a variety of neuroprotective effects, i.e. inhibition of NF-κB activity and prevention of NAD+ and ATP depletion^27^. On the other hand, increased *SNCA* expression is considered to be the culprit in PD pathophysiology. In contrast to VPA which has been used as an anticonvulsant and mood-stabilizer in a great number of patients over the years, no reports have associated the use of Emo with Parkinsonism (yet). Given the relatively low frequency of the incidence of parkinsonian symptoms observed with VPA (which are reversible upon cessation of VPA), one may assume that also Emo carries only a minor risk for parkinsonism and that none of the FDA approve compounds will affect *SNCA* expression to an extent which could increase the risk of PD^28^.

## Methods

### Cultivation and cell treatments of human neuroblastoma cell lines

The human SH-SY5Y neuroblastoma cell line was purchased from the European Collection of Authenticated Cell Cultures (ECACC) and used for the generation of our screening cell lines. All cell lines were cultivated in DMEM F12 Glutamax (Gibco) supplemented with 1% penicillin/streptomycin (Gibco) and 10% inactivated FBS (Sigma-Aldrich), respectively. For detachment, cells were treated with 1% Trypsin–EDTA (Gibco) for 10 min at 37 °C. All compounds were purchased from Selleckchem.

### Generation of *SNCA*-*GFP*-*LUC* fusion cell line via CRISPR/Cas9 gene editing and homologous recombination

CRISPR target sites for *SNCA* exon 6 were selected from the web tool chopchop (https://chopchop.cbu.uib.no/)^29,30^ using the genomic sequence of exon 6 of *SNCA* (NG_011851.1) and cloned into GeneArt CRISPR Nuclease Vector Kit (Thermo Fisher Scientific) according to manufacturer’s protocol. Primers for *SNCA*_CRISPR target site 1 Exon 6 as follows: forward TGGGAGCAAAGATATTTCTT**GTTTT**, reverse AAGAAATATCTTTGCTCCCA**CGGTG**.

For cloning of the homologous recombination (HR) vector HR150PA-1 (PrecisionX HR Targeting Vectors, System Bioscience), primers for the HR arms were tagged with 5′ palindromic sequences for respective restriction enzymes (bold) and amplified with Herculase II Fusion DNA Polymerase (Agilent) from genomic DNA (gDNA) of SH-SY5Y. Primers were as follows: left HR arms upstream *GFP*-*LUC* cassette (HR150PA-1), forward **GAATTC**GACATTCTGGCACAAGGGAATATCAG and reverse **GAATTC**GGCTTCAGGTTCGTAGTCTTGATACC, ***EcoRI***; for right HR arm downstream *GFP*-*LUC* cassette (HR150PA-1), forward **GGATCC**AATATCTTTGCTCCCAGTTTCTTGAG and reverse **GTCGAC**GACAGGATTGAAGGGAGAAATAGACC, ***BamHI*** and ***SalI***, respectively. Total length of HR arms were as follows: left HR arm 905 bp and right HR arm 889 bp. PCR products were sub-cloned into pJET1.2 (CloneJET PCR Cloning Kit, Thermo Fisher Scientific) and finally inserted into the HR150PA-1. Vector integrity was confirmed by sequencing. All restriction enzymes were fast digest enzymes and purchased from Fermentas, Thermo Fisher Scientific.

### Transfection, selection and screening of the *SNCA*-*GFP*-*LUC* knock-in cell line

Transfection of the CRISPR/Cas9- and the HR-plasmids, were performed with the Roti-Fect PLUS (Roth) transfection mix, according to manufacturer’s protocol. Selection pressure was applied after 24 h and maintained for 1–2 weeks. Single colonies were picked by using Corning Cloning Cylinders according to manufacturer’s protocol. Plates were duplicated when cells reached 80% confluency and protein lysates were generated to screen clones via western blot.

### Isolation of nucleic acids

#### Genomic DNA (gDNA)

Pelleted cells were incubated with 350 µl TENS buffer (50 mM TrisCl pH 8.0, 100 mM EDTA pH 8.0, 100 mM NaCl, 1% SDS) and 17.5 µl Proteinase K (10 mg/ml) overnight in a water bath at 55 °C. At the next day, 150 µL NaCl solution (saturated in H_2_O) were added, samples were incubated on ice for 5 min and centrifuged for 30 min. The supernatant was transferred into a fresh reaction tube, mixed with 500 µl isopropanol and incubated for 10 min at room temperature (RT). Samples were centrifuged for 30 min, supernatant was discarded and gDNA pellets were washed with 70% ethanol followed by 15 min centrifugation. Air-dried DNA was resolved in 10 mM Tris pH 7.5. All centrifugation steps were carried out at 16,000 rcf and 4 °C (adapted from “The Jacks Lab: DNA Isolation from Tail-Proteinase K Method”).

#### Miniprep/Maxipreparation

We used Top10F’ *E. coli* cells for transformation and mini-and maxi preparation ZR Plasmid Miniprep—Classic (Zymo Research) and the NucleoBond Xtra Maxi kit (Macherey-Nagel) were used, respectively.

### PCR

#### Standard PCR and gel-electrophoreses

For the generation of the homologous recombination arms 100–200 ng DNA was amplified in a total volume of 20 μl. Mastermix was prepared at final concentrations of 1 × reaction buffer (BioTherm, Genecraft), 250 µM dNTPs (Thermo Fisher Scientific), 0.2 µM of each primer, 1 unit Taq DNA polymerase (BioTherm, Genecraft) and filled up to 20 µl with H_2_O. After initial denaturation at 94 °C for 3 min PCR were run for 30 cycles (denaturation 94 °C for 30 s, annealing for 30 s at respective temperature, extension 68 °C for 1 min). PCR products were amplified in the Biometra TADVANCED thermocycler and separated in 1% TBE agarose gel containing 2.5 × GelRed Nucleic Acid Gel Stain (Biotium) for visualization. For the validation of *SNCA-GFP-LUC*-fusion, mRNA was converted into cDNA and amplified by using *SNCA*qF2 GGACCAGTTGGGCAAGAATG and HR150*GFP* reverse TGTCACGATCAAAGGACTCTGG primer. As a negative control for the wildtype locus we used *SNCA*qF2 and the corresponding reverse primer *SNCA*qR2 GGCACATTGGAACTGAGCAC.

### RT-qPCR assays

#### *LUC* mRNA

Total RNA for initial RT-qPCR assays was isolated with the Qiagen FastLane Cell RT-PCR SYBR Green Kit. Cells were seeded at a density of 8 × 10^4^ in 50 µl/well and 96 well format and treated the next day at effective compound concentration of 25 µM and equivalent DMSO controls. Each plate contained 30 compounds in triplicates and six DMSO controls. Cells were lysed in a total volume of 50 µl cell processing mix in accordance to manufacturer’s protocol with the following adaptions: (1) prolonged incubation (10 min) of the processing mix and (2) additional incubation of the lysates at 75 °C prior RT-qPCR (5 min). The RT-qPCR assays were performed with the QuantiTect SYBR Green RT-PCR Kit (Qiagen) in 384 well format. We used 3 µl of the cell processing mix (total of 50 µl) for amplification. Reactions were run in a Roche LightCycler 480 system. Primers were as follows: *LUC*, forward GAACATCACGTACGCGGAAT and reverse GCGCAACTGCAACTCCGATA. *LUC* mRNA expression was normalized to ubiquitin C (*UBC*) and glucuronidase beta *GUSB* housekeeping genes.

#### *SNCA* mRNA

For hit validation SH-SY5Y wildtype cells were treated at effective concentrations of 25 µM (12.5 µM for clomiphene-citrate) for 24 h in 24 well plate format and triplicates. Total RNA was extracted with the RNeasy Mini kit (Qiagen). RT-qPCR reactions were performed with the QuantiTect SYBR Green RT-PCR Kit (Qiagen) in a 96 well format and run in the Applied Biosystems HT7500 cycler. We used 50 ng of total RNA for amplification. Primers were as follows: *SNCA*, accession number NM_000345, purchased from Qiagen (Hs_SNCA_1_SG QuantiTect Primer Assay (QT00035903). *SNCA* mRNA expression was normalized to *UBC,* hypoxanthine phosphoribosyl-transferase 1 (*HPRT1*) and *GUSB* housekeeping genes.

Housekeeping primers were as follows: *HPRT1*, accession number NM_000194.3, forward TGACACTGGCAAAACAATGCA and reverse GGTCCTTTTCACCAGCAAGCT. *UBC*, accession number M26880, forward ATTTGGGTCGCGGTTCTTG and reverse TGCCTTGACATTCTCGATGGT^31^. *GUSB*, accession number XM_005250297.4, forward CCAGCGTGGAGCAAGACA and reverse CCATTCGCCACGACTTTGTT. Relative mRNA levels were calculated using the ΔΔCT Method for multiple housekeeping genes from Pfaffl, published in “A–Z of quantitative PCR”^32^.

### SDS PAGE and western blot analysis

For SDS-PAGE cells were harvested and lysed in RIPA buffer (50 mM TrisCl pH 7.5, 150 mM NaCl, 10 mM MgCl_2_, 0.5% Triton X 100) supplemented with Halt Protease Inhibitor-Cocktail (1 × final concentration) (Thermo Fisher Scientific) and 0.5 µl/ml benzonase (Merck) for 30 min on ice. Lysates were mixed with 4 × Laemmli loading buffer (200 mM TrisCl pH 6.8, 8% SDS, 6% β-mercapto-ethanol, 33% glycerol, spatula tip bromophenol blue) to a final concentration of 1 × and boiled for 10 min at 95 °C. Samples were loaded onto 15% SDS-PAGE gels.

### Nuclear extraction for histone Western blots

Nuclear extraction was performed according to Schreiber et al.^33^ with the following adaptions: Buffer C was supplemented with 0.1% SDS. Buffer A and C were supplemented with Halt Protease Inhibitor-Cocktail 1 × final concentration (Thermo Fisher Scientific). We used 200 µl of buffer A and 60 µl of buffer C (12-well plate format). Nuclei were sonicated for three seconds and three intervals at 50% power (Bandelin Sonopuls, HD2070, SH70G, type MS72), incubated on ice for 30 min and clarified by centrifugation. Supernatants were transferred to fresh tubes and stored at − 80 °C. All centrifugation steps were carried out for 10 min at 16,000 rcf and 4 °C.

### Western blot

Proteins were blotted onto methanol activated polyvinylidene difluoride (PVDF) membrane (GE Healthcare, Amersham Hybond), blocked with 5% milk-powder (Roth) in 1 × PBS-TWEEN 20 0.1% (PBST 0.1%) for 1 h and incubated with SNCA 2F12 (1:2000, MABN1817, Sigma-Aldrich) and beta actin (1:10,000, A5441, Sigma-Aldrich) primary antibodies overnight at 4 °C. Secondary HRP conjugated anti mouse antibody (1:4000, P0447, Dako) was applied for 1 h at room temperature. For histone H3 and H4 global acetylation (# 06-599 and # 06-598, Millipore) and H3K4 tri-methylation (C42D8, Cell Signaling Technology) were used. For normalization of nuclear extracts, we used the Lamin B1 antibody (D4Q4Z, Cell Signaling Technology). Secondary HRP conjugated anti rabbit antibody (1:4000, 7074 V, Cell Signaling Technology) was applied.

Membranes were washed three times with PBST 0.1% (1 × TBS-TWEEN 20 0.1% for histone Western blots) and imaged with enhanced chemiluminescence (ECL) in the ChemoCam imager (Intas). Signals were quantified with the ImageJ software^34^.

For SNCA and beta actin the secondary IRDye 800CW Donkey anti-Mouse IgG antibody (1:4000, LI-COR) was applied for 1 h at room temperature (in the dark). Membranes were imaged with the LI-COR Odysseys Clx (Model 9140; S/N CLX-0554) and signals were quantified using the Image Studio software 4.0.21. Treated cells were normalized to actin and DMSO control, respectively.

### In-Cell Western

For In-Cell Western (ICW) experiments, cells were plated in 96 well plates (black/clear, Falcon) at a density of 8 × 10^4^ cells/well. Cells were treated at the next day. After treatment, media were discarded and cells were fixed with 100 µl ice cold 100% methanol (− 20 °C) for 15 min at RT on an orbital shaker. Methanol was discarded, permeabilized cells were washed with 100 µl 1 × PBS and blocked with 0.5% casein blocking solution (Casein diluted in 1 × PBS) for 30 min at RT. After blocking, cells were incubated with 50 µl of alpha synuclein 2F12 primary antibody dilution (diluted 1:2000 in 0.5% casein PBS + 0.1% blocking solution (PBST)) at 4 °C on an orbital shaker overnight. On the next day, cells were washed 3 × with 100 µl 1 × PBST and incubated with 50 µl of CellTag 700 Stain (1:1000 from LI-COR) and secondary IRDye 800CW Donkey anti-Mouse IgG antibody (1:1000, from LI-COR) in 0.5% casein PBST blocking solution for 1 h at RT on an orbital shaker. Plates were protected from light. Cells were washed 3 × with 100 µl PBST and 1 × with PBS and imaged with the LI-COR Odyssey Clx (Model 9140; S/N CLX-0554) and signals were quantified using the Image Studio software 4.0.21.

For analysis the Image Studio 4.0 software (provided from LI-COR) was used and signals were normalized to CellTag700 and DMSO, respectively. As a background control, cells were incubated with secondary antibody and CellTag700 alone.

### *LUC* assay

Bioactive compound collections (Selleckchem) were randomly spotted—initially at a concentration of 10 µM in three independent experiments. We used valproic acid (VPA), a known modulator of α-syn expression, as a positive control (Fig. 2A).

The screening process was fully automated. For the luciferase assay 2 × 10^4^ cells/well in a volume of 30 µl were seeded into nunc white 384 well plates (Thermo Fisher Scientific). Cells attached and grew for approx. 18 h at 37 °C before treatment. The pre-spotted 384 well compound plates (100 nl/well) were diluted with 25 µl medium/well and shaken for 5 min with 1200 rpm at RT. Subsequently, 10 µl of the compound dilution were applied to 384 well cell plates, resulting in a final concentration of 10 µM and incubated for 24 h at 37 °C. Controls were distributed on the assay plate in a fixed layout for all three independent experiments. The tested drugs were randomly distributed for the three experiments to avoid well location dependent effects (Fig. 2A). Cells were lysed by adding 40 µl of ONE Glo (Lysis Buffer and Luciferase Substrate, Promega) to each well (on top of medium), incubated for 5 min while shaking at 1200 rpm and luciferase signal was measured with the Paradigm Reader at 1200 ms integration time.

For hit definition the luciferase signal of treated cells was normalized to untreated controls per plate. Compounds showing an increased (activators) or decreased (inhibitors) luciferase signal of more than the four-fold standard deviation of the mean (SD) of untreated controls were considered as effective modulators (Fig. 2B).

Repeated experiments were conducted in Nunc white 96 well plates and measured with the Centro LB 960 (Berthold Technologies) at 1200 ms integration time.

### Cell viability tests

To screen for potentially cytotoxic effects we performed a combination of a homogenous resazurin test and an image-based high content screen (HCS) on single cell level in a dose range of 0.25–40 µM, respectively. The resazurin assay was performed according to manufacturer’s protocol. For the HCS, the nuclei of treated cells were stained with the fluorescent DNA probe DRAQ5. After imaging, living and dead cells were counted per well and total cell viability were calculated for each well and applied compound concentration.

### Statistics

We used one-way ANOVA (α = 0.05) followed by the recommended Dunnett’s multiple comparison test to check for statistical significance. All statistical analyses were performed in GraphPad Prism 7.02.

## Supplementary Information

Supplementary Figures.
