# Supplementary material for: Activators of alpha synuclein expression identified by reporter cell line-based high throughput drug screen
Source: Sci Rep. 2021 Oct 6;11:19857. doi: 10.1038/s41598-021-98841-9 (PMC8494889; doi:10.1038/s41598-021-98841-9)
Supplement: Supplementary file 1 — Supplementary Figures. [file 41598_2021_98841_MOESM1_ESM.pdf]

# ACTIVATORS OF ALPHA SYNUCLEIN EXPRESSION IDENTIFIED BY REPORTER CELL LINE-BASED HIGH THROUGHPUT DRUG SCREEN


Fabian Stahl[1,2], Philip Denner[1], Dominik Piston[1], Bernd O. Evert[2], Laura de Boni[2], Ina Schmitt[2], Peter Breuer[2]*, Ullrich Wüllner[1.2]*

[1]DZNE, German Center for Neurodegenerative Diseases, Germany
[2]University of Bonn, Department of Neurology, 53105, Bonn, NRW, Germany

**Corresponding authors:**

Prof. Dr. med. Ullrich Wüllner; PD Dr. rer. nat. Peter Breuer

DZNE & University Hospital Bonn, Department of Neurology

Venusberg-Campus 1

53105 Bonn, NRW, Germany

Tel: +49 228 2871 5714

Fax: +49 228 2871 5024

Email: ullrich.wuellner@dzne.de, peter.breuer@ukbonn.de

# Supplementary Fig. S1

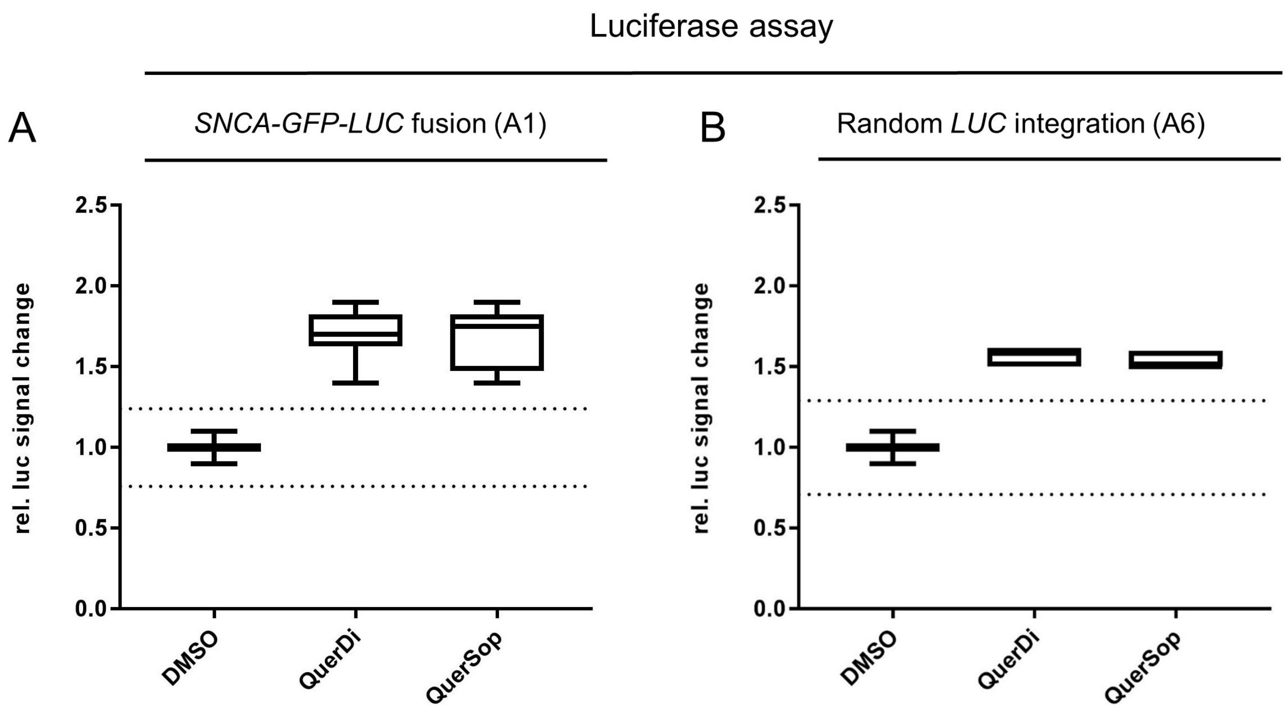


**Supplementary Fig. S1. LUC assay in the screening (A1) and control cell line (A6) for activators of α-syn.** (**A**) Signal fold-change in the LUC assay of A1 and (**B**) A6 cell lines. Luciferase signal change was determined by normalizing six replicates of treated cells to DMSO. Boxplot diagrams represent 5-95 percentile. Dotted lines depict threshold of the four-fold standard deviation of DMSO controls. Compounds were applied at a final concentration of 25 µM for 24 h.

# Supplementary Fig. S2

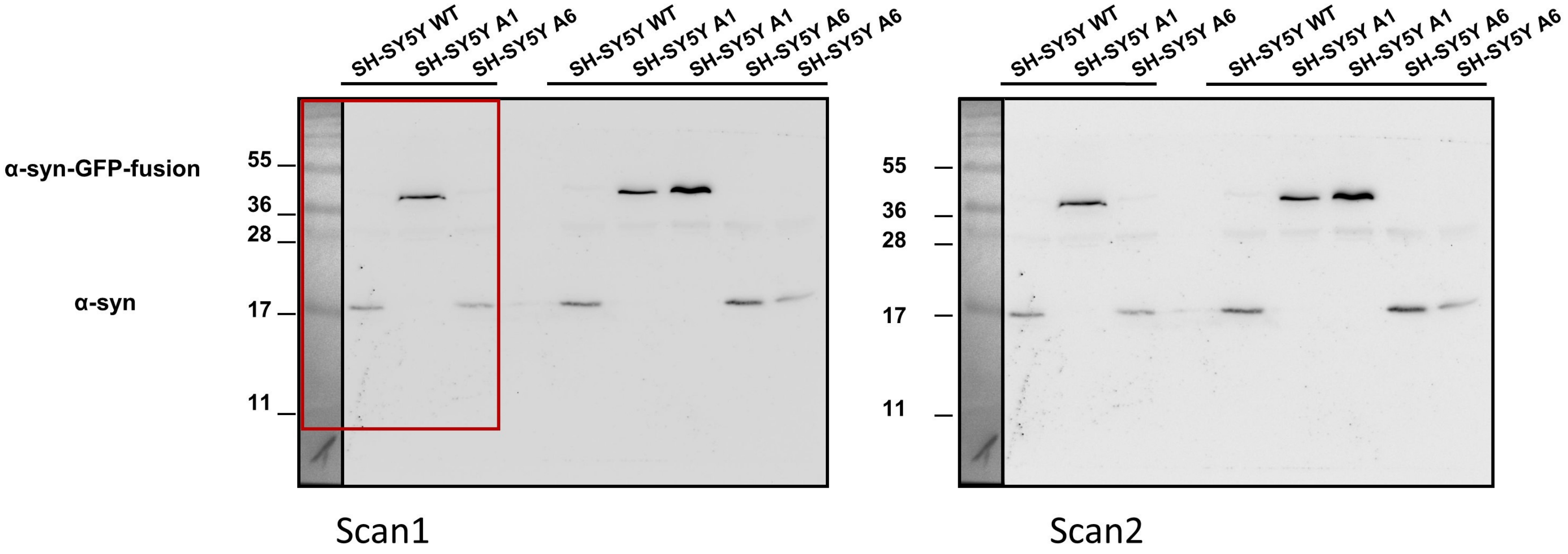


**Supplementary Fig S2. Sequentially exposed images of full-size WB membrane used in Fig. 1 with higher background signal.**
Black box indicates the full-size WB shown in Fig 1. Red box indicates Fig. 1 of the main text.

# Supplementary Fig. S3

**Supplementary Fig S3**. **Example in-cell Western from compound treatment of SH-SY5Y wild type cells (primary data).** (**A**) SNCA 2F12 (1:2,000, MABN1817, Sigma-Aldrich) and secondary IRDye 800CW Donkey anti-Mouse IgG antibody (1:4,000, LI-COR). (**B**) CellTag 700 Stain (1:1,000, LI-COR). (**C**) Overlay of (**A**) and (**B**). (**D**) Quantification of each well/row normalized to CellTag 700 and DMSO control. The means and standard deviations (SDs) of each rows are depicted ( n=6; outliers were excluded). 1) empty well control, 2) non-treatment control (NTC), 3) DMSO, 4) Clo (12.5 µM), 6) Con (25 µM), 7) Emo (25 µM), 8)** QuerDi (25 µM), 9)** QuerSop (25 µM), 11) VPA (600 µM), 12) background control secondary antibody. *Compounds used in lane 5 and 10 were cytotoxic. **QuerDi and QuerSop were not described in the main manuscript since they show also an increase in LUC signal in the A6 specificity control cell line (see Supplementary Fig. S1). ns. = non-significant. p=0.0001. ICWs were imaged with the LI-COR Odysseys Clx (Model 9140; S/N CLX- 0554) and signals were quantified using the Image Studio software 4.0.21.

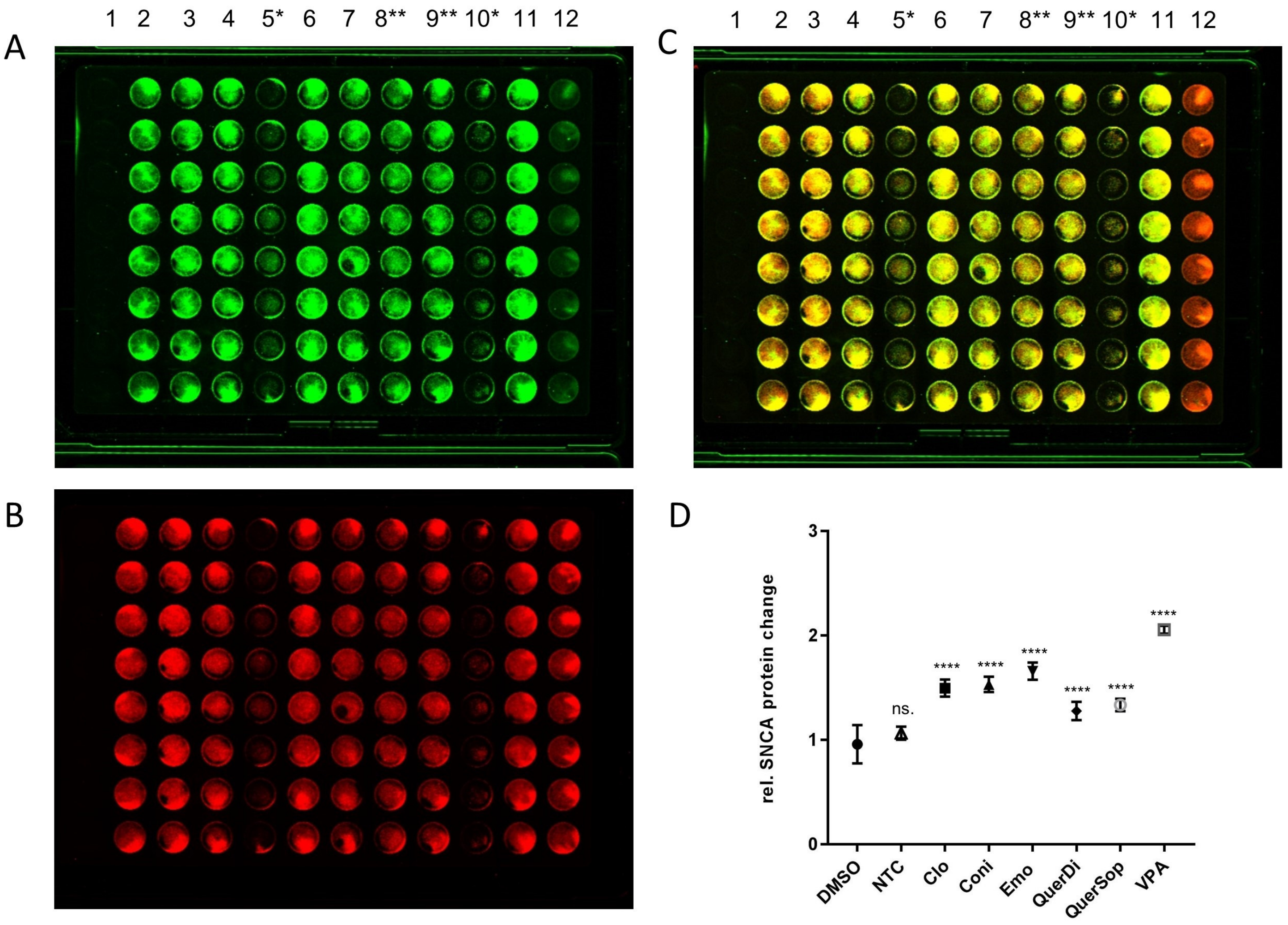

Supplementary Fig. S4

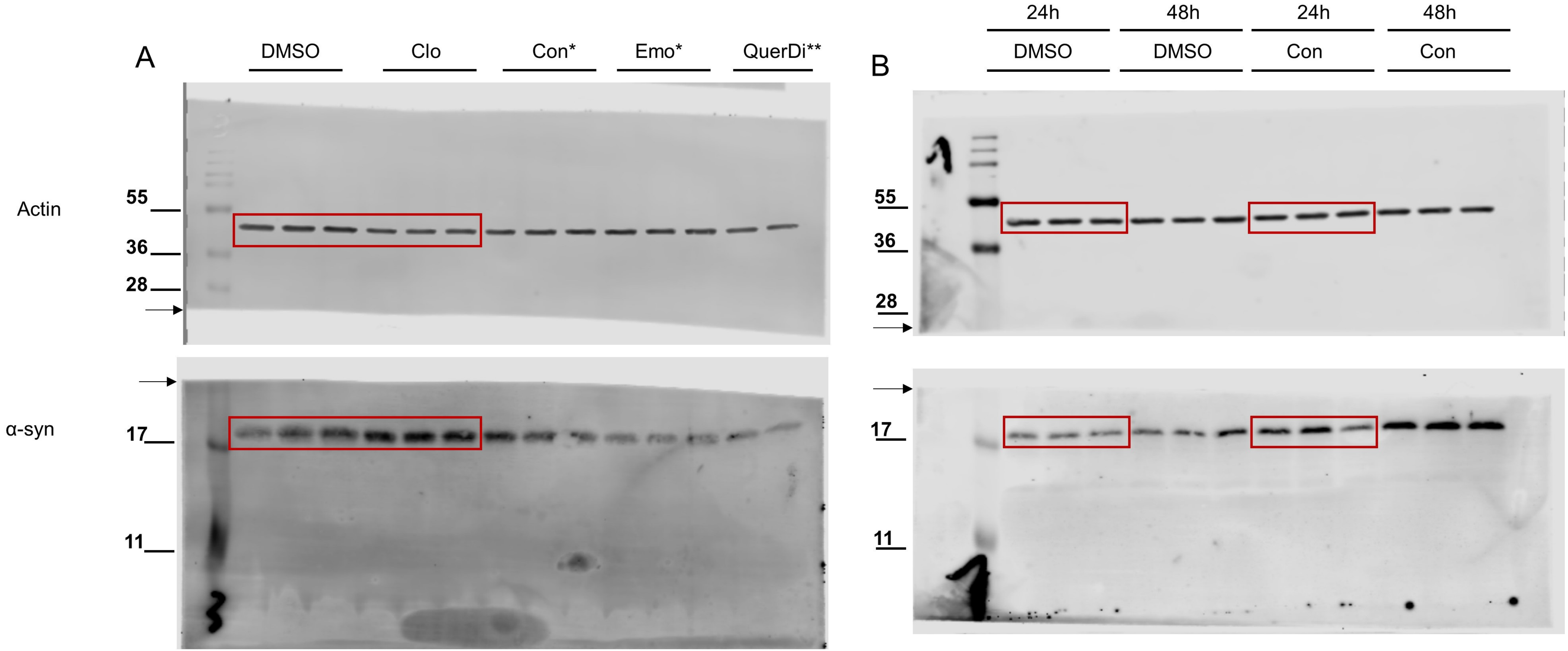


**Supplementary Fig. S4. Compound treatment induces α-syn protein levels.** Full-size WB membranes of treated SH-SY5Y wildtype cells. Membranes were cut in two halves (black arrow) to incubate with actin (A5441, upper) and α-syn (MABN1817, lower) antibodies in parallel. Compounds were applied at final concentrations of 25 µM (Clo 12.5 µM) for (**A**) 24 h and (**B**) 24 h or 48 h. (**A**) *low α-syn levels were due to lower blotting efficiency. **Quercetin Dihydrate (QuerDi) was found to be an activator in the luciferase assay but also increased LUC signal in the A6 control cell line. Therefore, it was not described in the main manuscript (see Supplementary Fig. S1). We used (**A**) DMSO and Clo and (**B**) DMSO 24 h and Con 24 h for Fig. 4 (red boxes). WB membranes were imaged with the LI-COR Odysseys Clx (Model 9140; S/N CLX- 0554) and signals were quantified using the Image Studio software 4.0.21.

# Supplementary Fig. S4

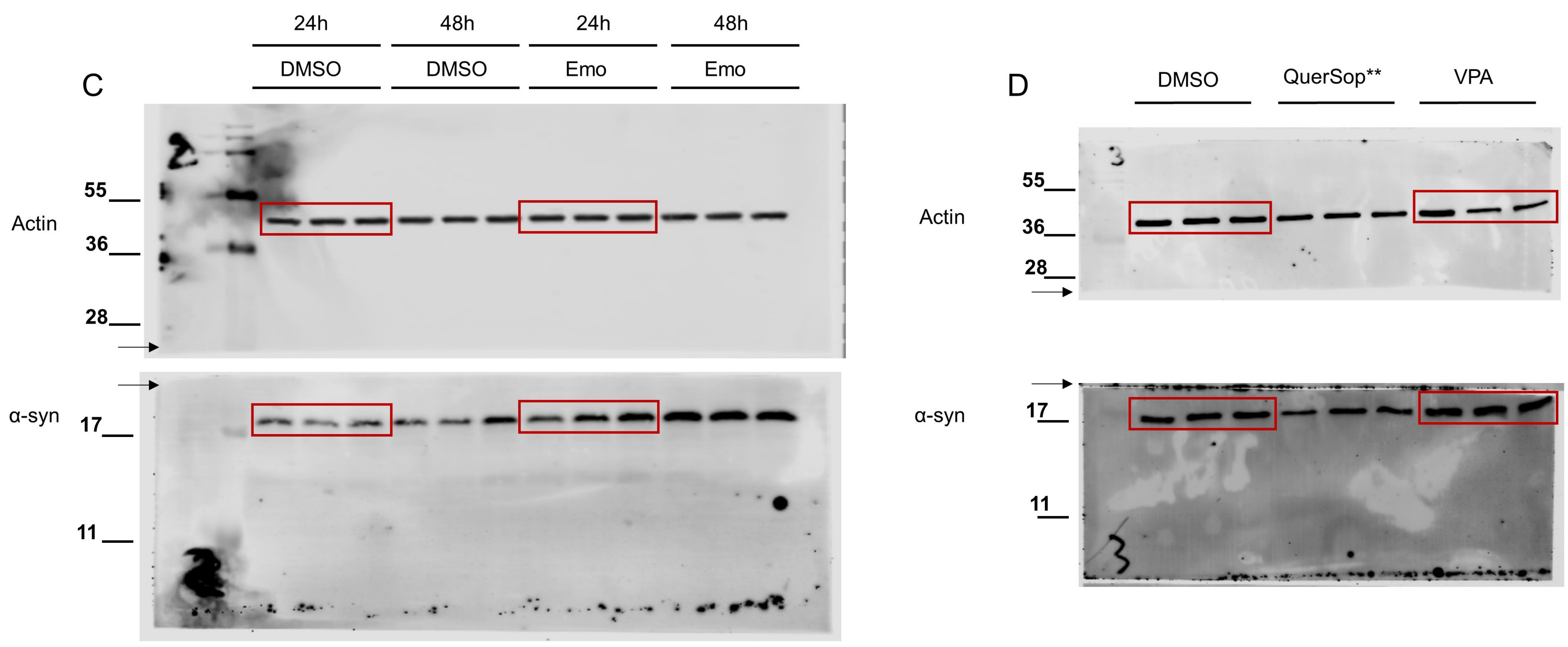


**Supplementary Fig. S4. Compound treatment induces α-syn protein levels.** Full-size WB membranes of treated SH-SY5Y wildtype cells. Membranes were cut in two halves (black arrow) to incubate with actin (A5441, upper) and α-syn (MABN1817, lower) antibodies in parallel. (**C**) Emo was applied at final concentration of 25 µM for 24 h and 48 h. (**D**) Compounds were applied at final concentrations of 25 µM and VPA 600 µM. **Quercetin Sophoretin (QuerSop) was found to be an activator in the luciferase assay but also increased LUC signal in the A6 control cell line. Therefore, it was not described in the main manuscript (see Supplementary Fig. S1). We used (**C**) DMSO 24 h and Emo 24 h and (**D**) DMSO and VPA for Fig.4 (red boxes). WB membranes were imaged with the LI-COR Odysseys Clx (Model 9140; S/N CLX-0554) and signals were quantified using the Image Studio software 4.0.21.

Supplementary Fig. S5

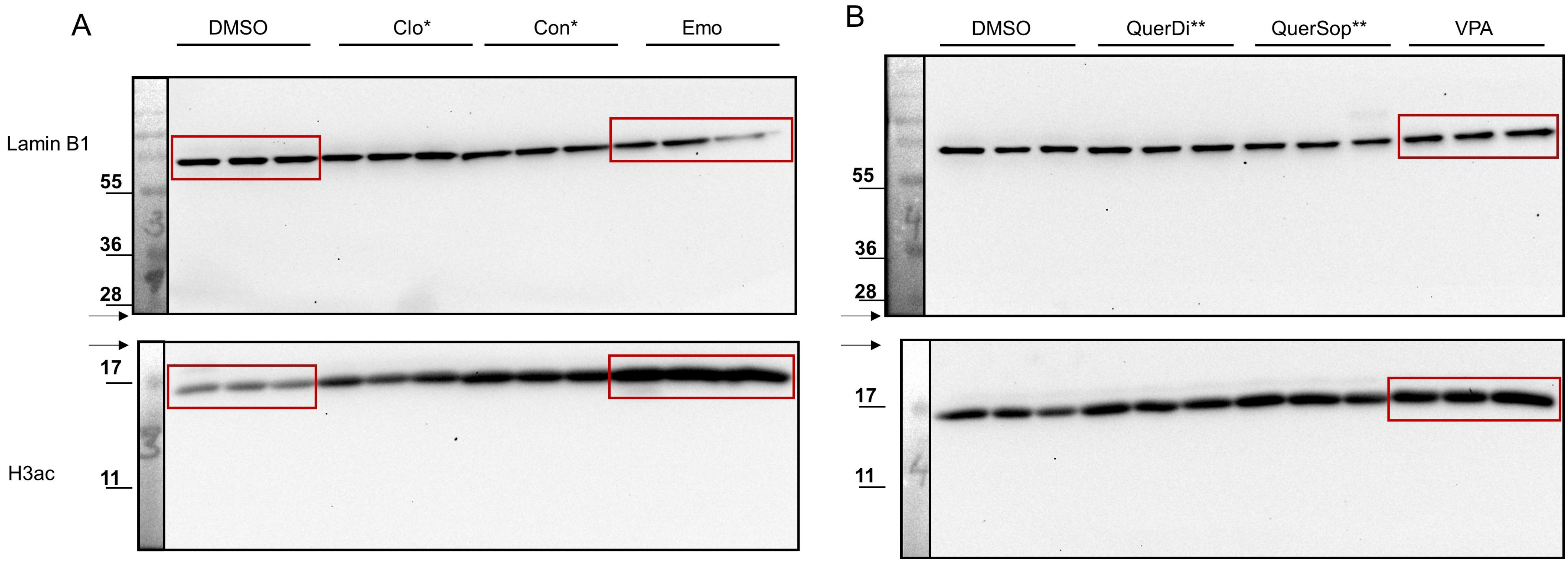


**Supplementary Fig. S5. Compound treatment increased histone H3ac levels.** Full-size WB membranes of treated SH-SY5Y wildtype cells. Membranes were cut in two halves (black arrow) to incubate with Lamin B1 (D4Q4Z, upper) and α-syn (MABN1817, lower) antibodies in parallel. (**A**) Compounds were applied at final concentrations of 25 µM (Clo 12.5 µM) for 24 h. (**B**) Compounds were applied at final concentrations of 25 µM (VPA 600 µM) for 24 h. *Clo and Con did not show a significant change in H3ac levels (see Supplementary Fig. S5 F). **Quercetin Dihydrate (QuerDi) and Quercetin Sophoretin (QuerSop) were found to be activators in the luciferase assay but also increased LUC signal in the A6 control cell line. Therefore, these compounds were not described in the main manuscript (see Supplementary Fig. S1). We used (**A**) DMSO and Emo and (**B**) VPA for Fig. 5 (red boxes).

# Supplementary Fig. S5

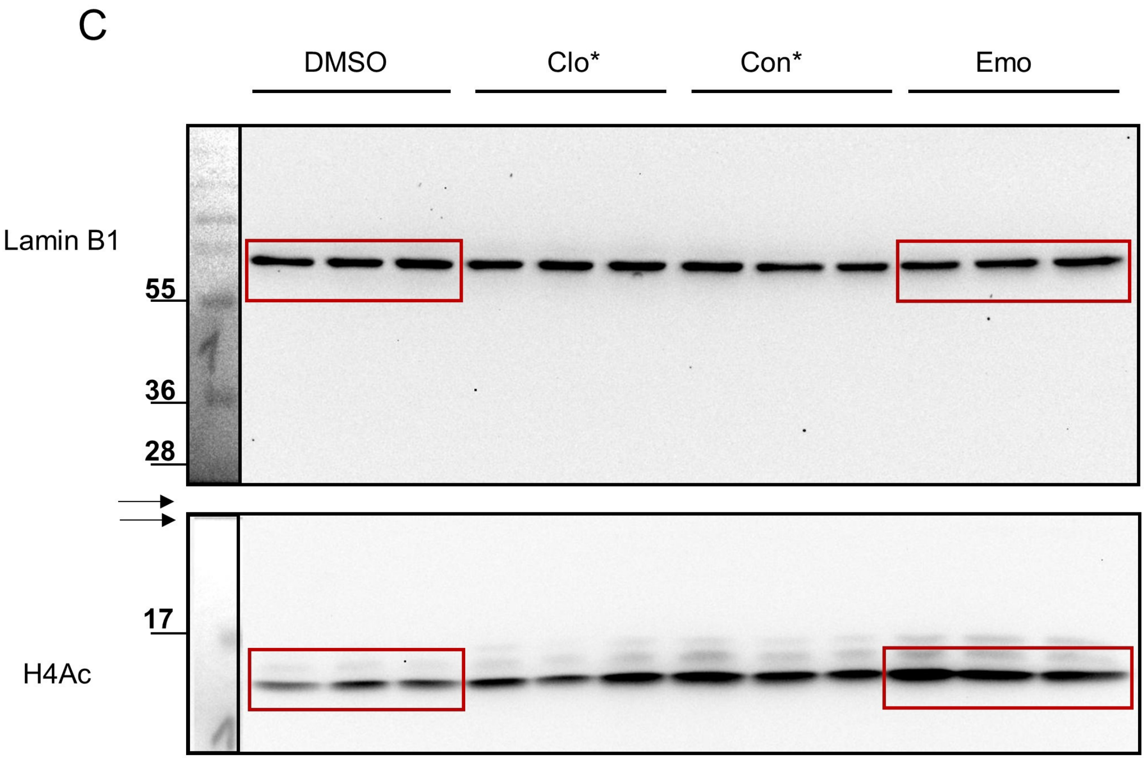


**Supplementary Fig. S5. Compound treatment increased histone H4ac levels.** (**C**) Full-size WB membrane of treated SH-SY5Y wildtype cells. The membrane was cut in two halves (black arrow) to incubate with Lamin B1 (D4Q4Z, upper) and α-syn (MABN1817, lower) antibodies in parallel. Compounds were applied at final concentrations of 25 µM (Clo 12.5 µM) for 24 h. *Clo and Con did not show a significant change in H4ac levels (see Supplementary Fig. S5 F). We used (**C**) DMSO and Emo for Fig. 5 (red boxes).

# Supplementary Fig. S5

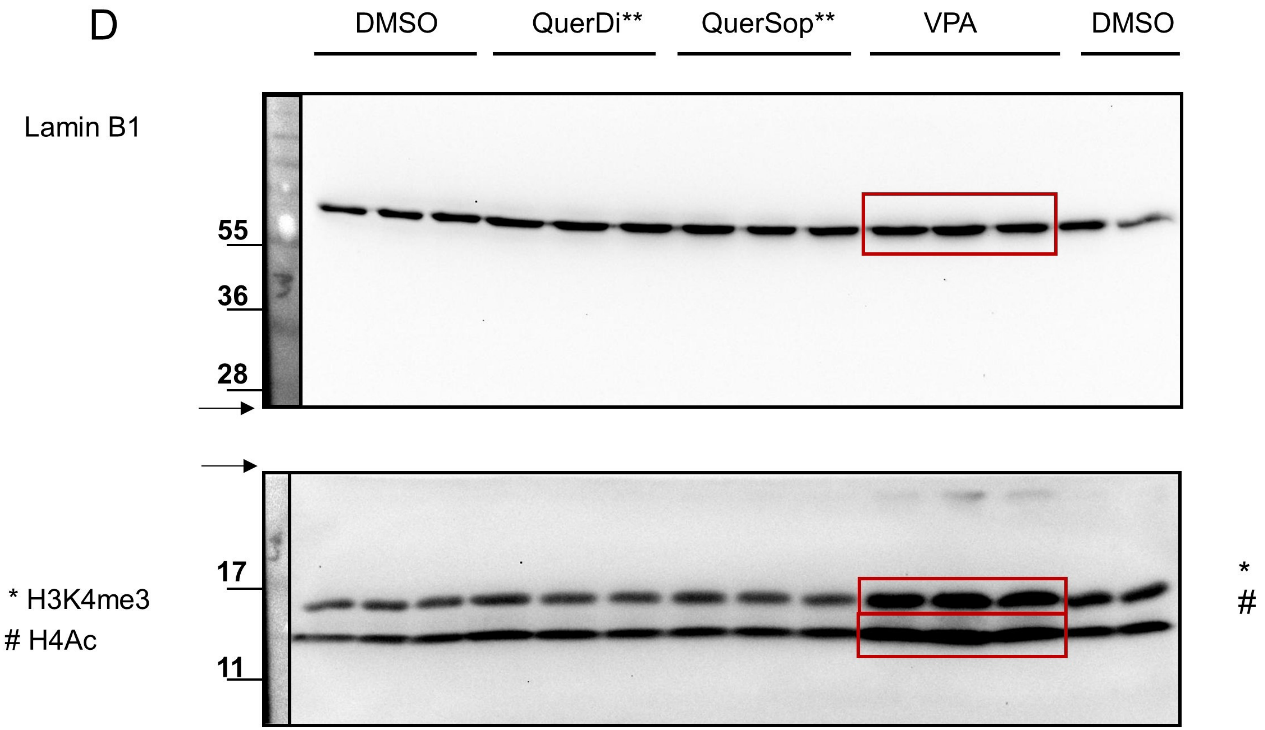


**Supplementary Fig. S5. Compound treatment increased histone H4ac and H3K4me3 levels.** (**D**) Full-size WB membrane of treated SH-SY5Y wildtype cells. The membrane was cut in two halves (black arrow) to incubate with Lamin B1 (D4Q4Z, upper) and α-syn (MABN1817, lower) antibodies in parallel. Compounds were applied at final concentrations of 25 µM (VPA 600 µM) for 24 h. **Quercetin Dihydrate (QuerDi) and Quercetin Sophoretin (QuerSop) were found to be activators in the luciferase assay but also increased LUC signal in the A6 control cell line. Therefore, these compounds were not described in the main manuscript (see Supplementary Fig. S1). We used VPA for Fig. 5 (red boxes).

# Supplementary Fig. S5

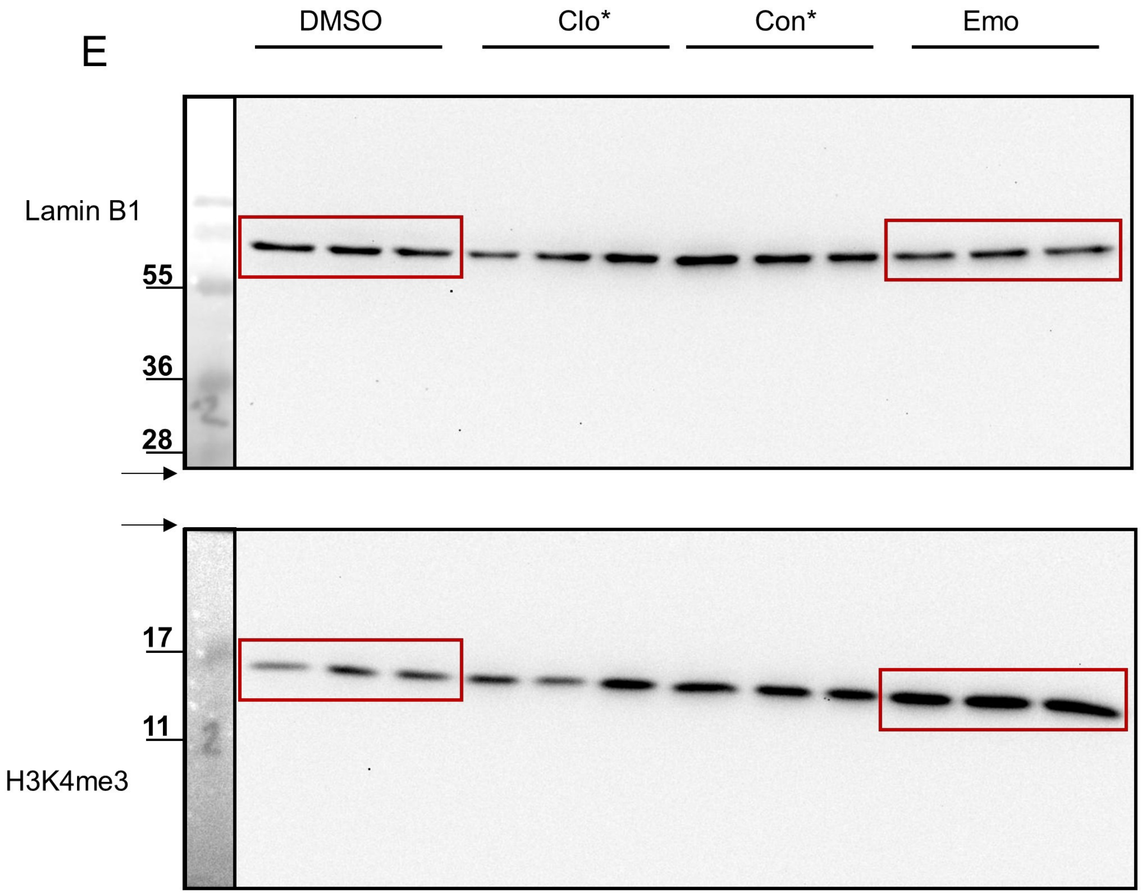


**Supplementary Fig. S5. Compound treatment increased histone H3K4me3 levels.** (**E**) Full-size WB membrane of treated SH-SY5Y wildtype cells. Membranes were cut in two halves (black arrow) to incubate with Lamin B1 (D4Q4Z, upper) and α-syn (MABN1817, lower) antibodies in parallel. Compounds were applied at final concentrations of 25 μM (Clo 12.5 μM) for 24 h. *Clo and Con did not show a significant change in H3K4me3 levels (see Supplementary Fig. S5 F). We used (**C**) DMSO and Emo for Fig. 5 (red boxes).

Supplementary Fig. S5

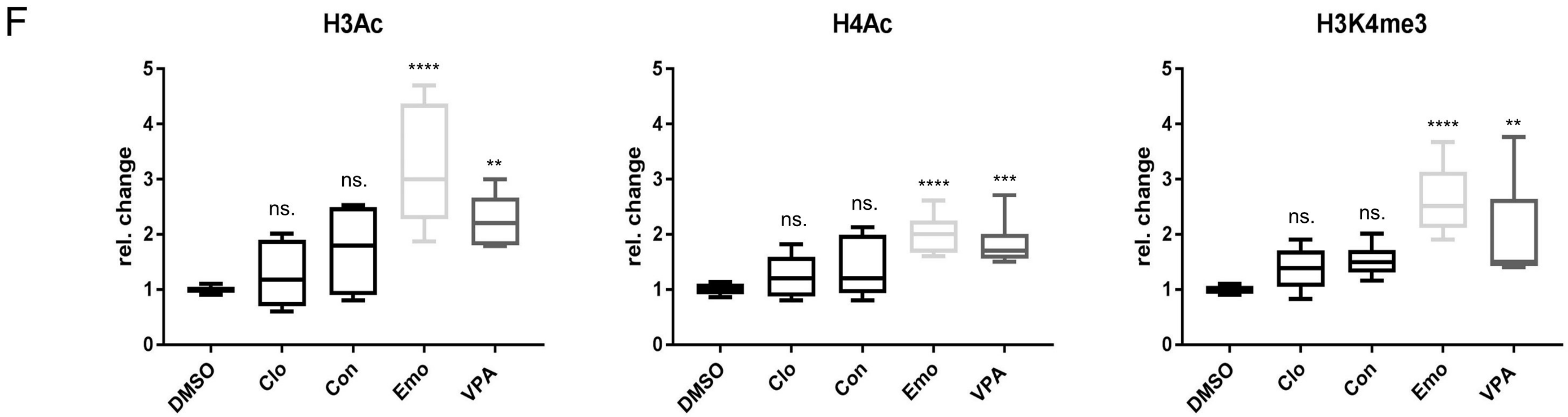


**Supplementary Fig. S5. (F) Treatment with Emo and VPA lead to increased H3/H4ac and H3K4me3 levels whereas Clo and Con showed no significant change in histone modifications.** Boxplot diagrams represent 5-95 percentile. Three independent experiments with three repetitions were conducted. Protein levels were determined by WB and normalized to Lamin B1 and DMSO, respectively. Compounds were applied at a final concentrations of 25 µM (Clo 12.5 µM) for 24 h. VPA was used as a positive control at a concentration of 600 µM. ns.= non significant. $^{**}p<0.01$, $^{***}p<0.001$ $^{****}p=0.0001$.

# Supplementary Fig. S6

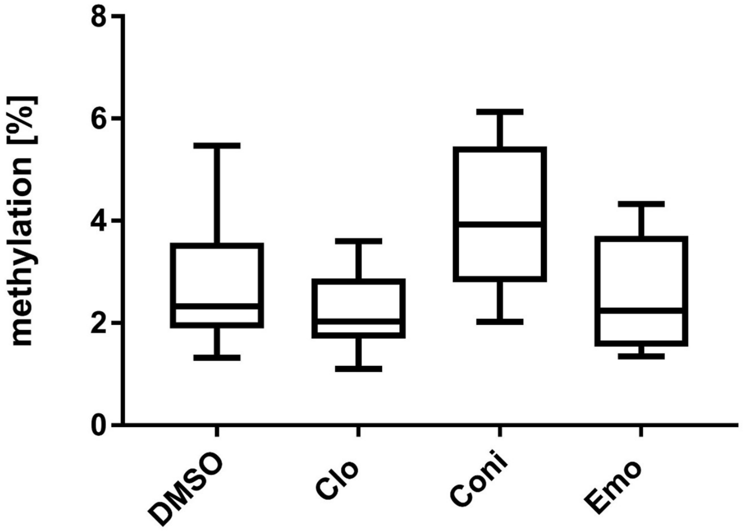


**Supplementary Fig. S6 Analysis of *SNCA* intron 1 methylation after compound treatment.** Overall methylation of CpGs 2-7 and CpGs 16-18 within the *SNCA* intron 1 region showed no significant modulation after compound treatment. Compounds were applied at final concentrations of 25 µM (Clo 12.5 µM) for 24h. Boxplot diagrams depict all tested CpGs in the different treatment conditions (sample size per condition: n=1). We used 500 ng gDNA for each bisulfite conversion per reaction (EZ-96 DNA Methylation-Gold Kit, Zymo Research). Amplification of the sequences containing CpGs 2-7 were performed with the HotStarTaq Plus Master Mix Kit (Qiagen) and primers as follows: forward: GGAGTTTAAKGAAAGAGATTTGATT; biotinylated reverse: Bio-CTCCYTACACTTCCATTTCATTATTTAC and for CpGs 16-18 we used forward primer: GTTTGGTAAATAATGAAATGGAAGTGTA; biotinylated reverse: Bio-CCAAATATAATAATTCTAATCCATC. Pyrosequencing was performed in PyroMark Q24 using sequencing primer as follows: for CpGs 2-7: GTTTTTTYGGGTGGTT and for CpGs 16-18 TTGAGAGATTAGGTTGTT.

Supplementary Fig. S7

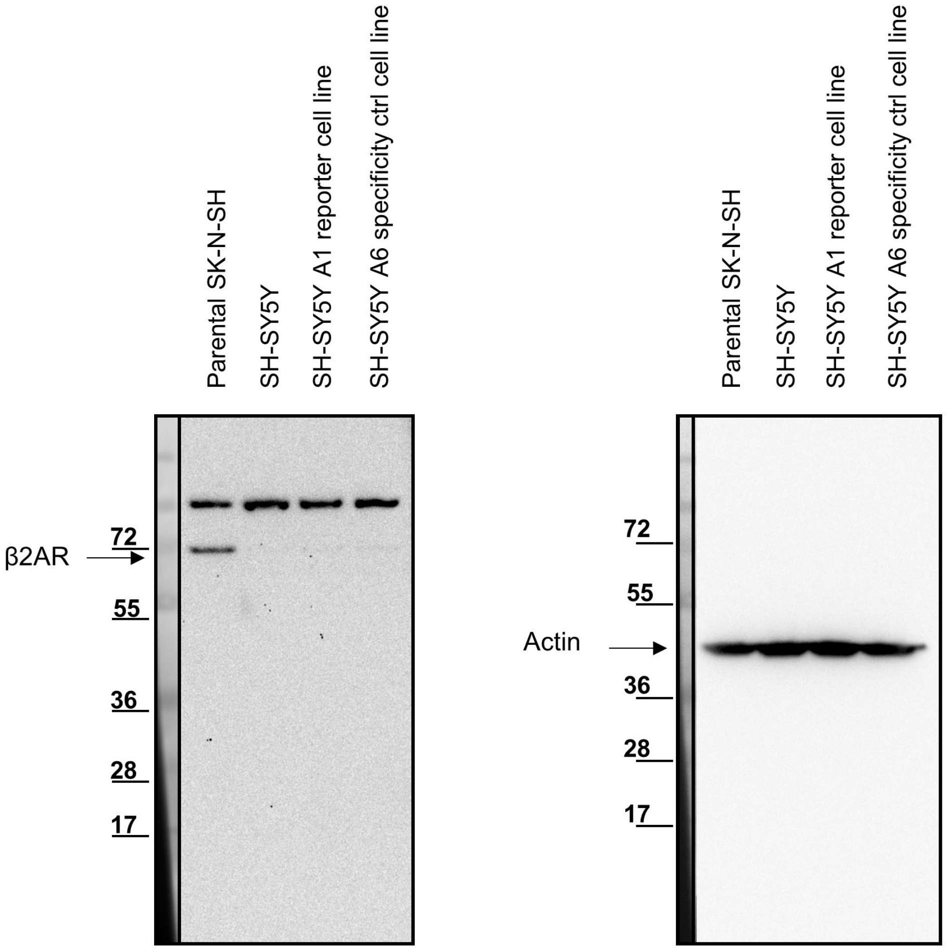


**Supplementary Fig. S7 Western blotting to determine β2AR expression in SH-SY5Y cells.** In the parental SK-N-SH the β2AR is clearly detectable (ab182136, abcam). However, no β2AR expression is detectable in SH-SY5Y cells and the generated A1 reporter cell line and A6 specificity control.

Supplementary Fig. S8

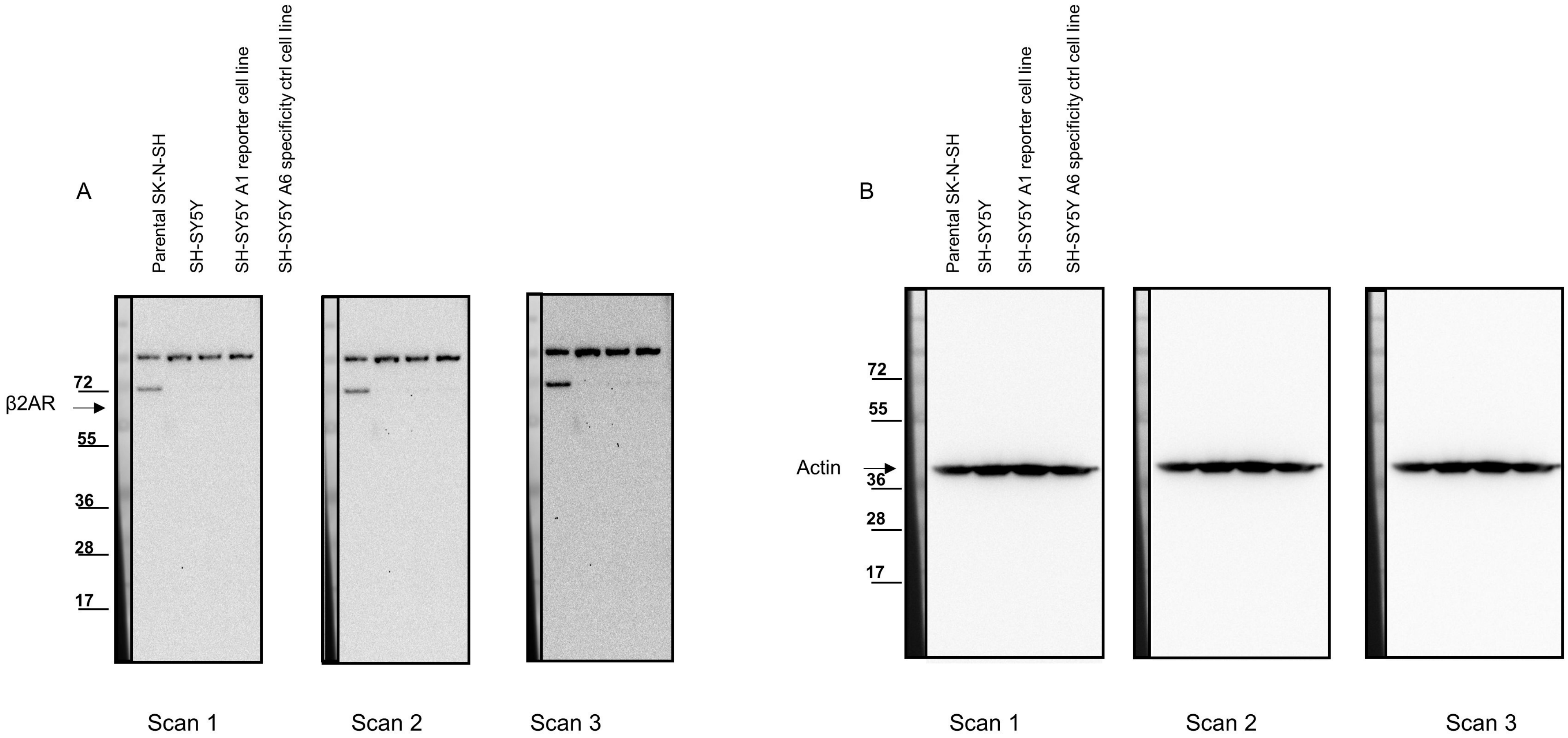


**Supplementary Fig. S8. Several exposure images of supplementary Fig. S7.** (**A**) β2AR- and (**B**) actin WB.

Supplementary Fig. S9

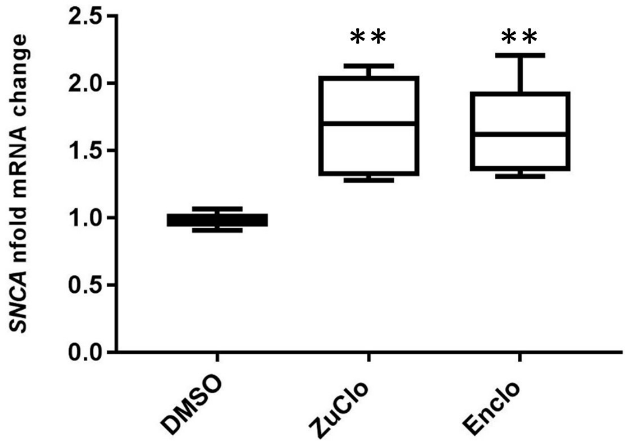


**Supplementary Fig. S9 Expression fold changes of *SNCA* mRNA measured with RT-qPCR after treatment with Clo isomers zuclomiphene (ZuClo) and enclomiphene (EnClo).** RT-qPCR data were normalized to two (*UBC*/*GUSB*) housekeeping genes and DMSO control, in duplicates, respectively. Three independent experiments were performed. Boxplot diagrams depict six measurements and represent 5-95 percentile. Compounds were applied at a final concentration of 10 μM for 24 h. **p<0.01.

Supplementary Fig. S10

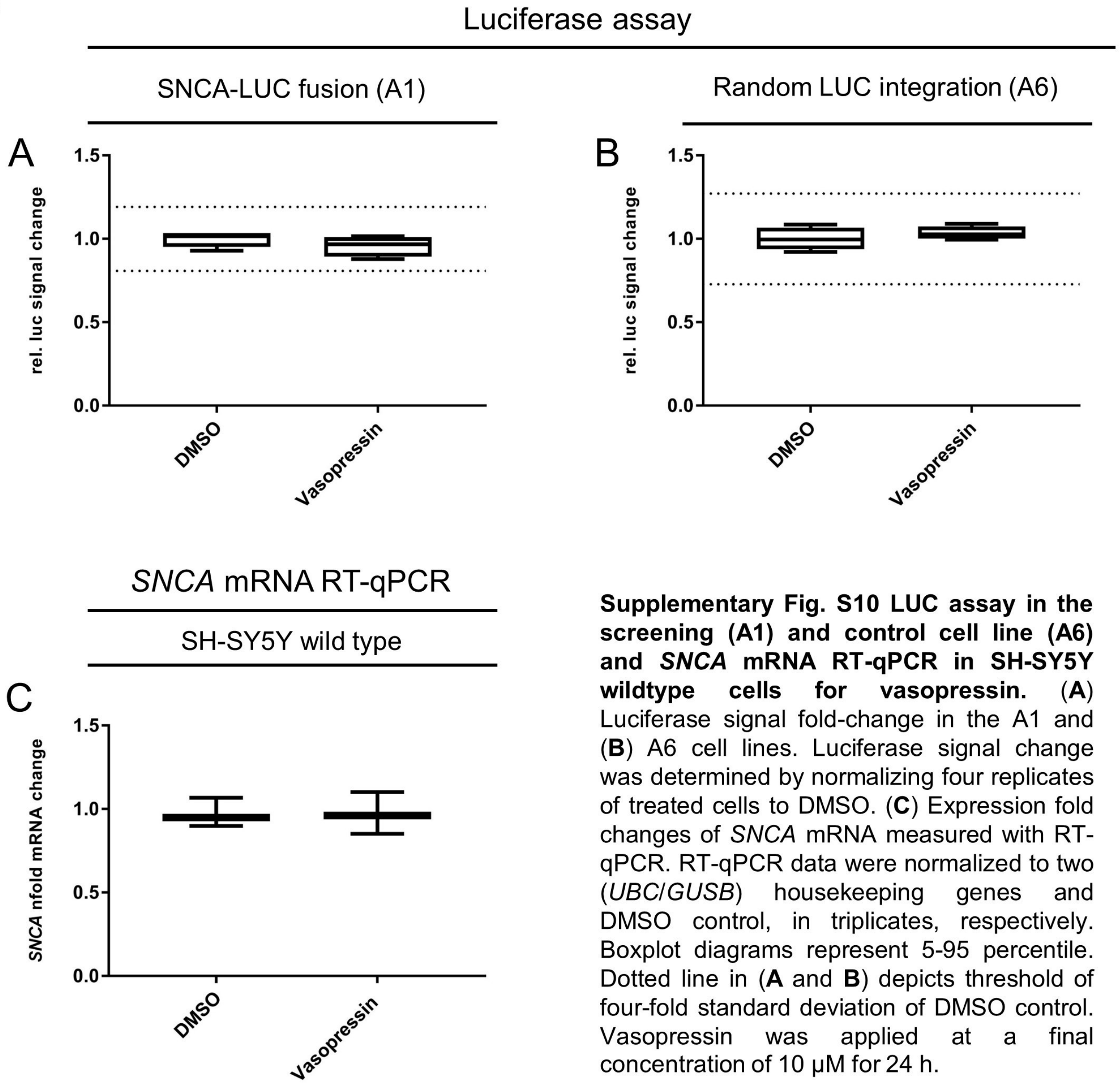


**Supplementary Fig. S10 LUC assay in the screening (A1) and control cell line (A6) and *SNCA* mRNA RT-qPCR in SH-SY5Y wildtype cells for vasopressin.** (**A**) Luciferase signal fold-change in the A1 and (**B**) A6 cell lines. Luciferase signal change was determined by normalizing four replicates of treated cells to DMSO. (**C**) Expression fold changes of *SNCA* mRNA measured with RT-qPCR. RT-qPCR data were normalized to two (*UBC*/*GUSB*) housekeeping genes and DMSO control, in triplicates, respectively. Boxplot diagrams represent 5-95 percentile. Dotted line in (**A** and **B**) depicts threshold of four-fold standard deviation of DMSO control. Vasopressin was applied at a final concentration of 10 µM for 24 h.
